# Differences in the swallowing process of newborns and healthy preterm infants: first results with a non-invasive bioimpedance and electromyography measurement system

**DOI:** 10.1007/s00405-023-08344-8

**Published:** 2023-11-24

**Authors:** Nicole Hübl, Benjamin Riebold, Dirk Schramm, Rainer O. Seidl

**Affiliations:** 1 Department of General Pediatrics, Neonatology and Pediatric Cardiology, Medical Faculty and University Hospital Düsseldorf, Heinrich Heine University Düsseldorf, Germany, Moorenstrasse 5, 40225 Düsseldorf, Germany; 2 TU Berlin, Control Systems Group, Einsteinufer 17, 10587 Berlin, Germany; 3 Ear-Nose and Throat, Unfallkrankenhaus Berlin, UKB, Warener Str.7, 12683 Berlin, Germany

**Keywords:** Preterm infant, Swallowing, Breathing, Aspiration, Neonate, Bioimpedance

## Abstract

**Purpose:**

Preterm infants (PI) have difficulty coordinating sucking, swallowing and breathing, and there is a risk of aspiration. The causes of this are not yet sufficiently understood. The aim of this study was to test a novel measurement device to measure breathing and pharyngeal processes involved in swallowing externally in everyday life to identify possible differences in neonates (NB) and PI.

**Methods:**

Forty healthy NB were studied at 4–8 weeks of age (mean: 6.7 weeks) and 20 healthy PI (mean gestational age 30.5 weeks) at postmenstrual age (PMA) 34/35 weeks (mean PMA 35.1 weeks) during a single feeding. Surface electrodes were used to measure bioimpedance and electromyography reflecting swallow-related changes in the pharynx and muscle activation of the tongue and submental muscles. A respiratory belt was combined with recording of the depth of chest movements and the occurrence of pauses in breathing.

**Results:**

Velocity and extent of pharyngeal closure did not differ significantly across the feeding period (velocity: *p*=0.09, closure: *p*=0.17), but during the first two suck–swallow bursts PI had greater velocity (*p*<0.001*) and extent of pharyngeal closure (*p*=0.004*) than NB. The duration of swallowing phases was significantly longer in PIs (*p*<0.001*), their muscle activation decreased faster (*p*<0.001*), and they had more pauses in breathing than NBs.

**Conclusions:**

The novel measurement device allowed, for the first time in everyday life, the measurement of factors influencing swallowing and breath–swallow coordination in NBs and PIs. PIs showed differences from NBs most likely due to differences in muscle strength and condition.

## Introduction

Feeding of newborns and preterm infants differs in several ways. Newborns are usually born with an age-appropriate healthy cardiorespiratory system, gastrointestinal functioning, motor control, and vigilance supporting their overall state regulation and feeding performance. During feeding they can suck efficiently from the breast or bottle by applying suction and expression components of sucking [1–4]. Newborn’s breathing is integrated into sucking bursts and is coordinated well with efficient swallowing, leading to a secure feeding with very limited risk of aspiration [5–7].

Preterm infants suffer from immaturity that can lead to poor cardiorespiratory and gastrointestinal functioning, decreased motor control and strength, and limited vigilance, resulting in less optimal preconditions for successful feeding performance. They fatigue early in their feeding trials, feed inefficiently, and are at risk of aspiration [8–12]. Nasogastric tube feeding is typically needed until hospital discharge or beyond [13] and prevalence of ongoing problematic feeding high [14].

A safe and efficient feeding process requires sufficient functioning of sucking, swallowing, and breathing and decent coordination of all three [4, 6, 9, 15, 16]. Preterm infants can improve their suction and expression components of sucking through postnatal maturation and feeding experience [9, 17]. Rommel and colleagues [18] reported on stable pharyngeal peak pressure amplitudes and stable velocity of pharyngeal peristalses from 32 to 36 week postmenstrual age (PMA). A slight age-related difference of pharyngeal pressure at laryngeal inlet was detected before 32 week PMA, as well as a decrease in time for upper esophageal sphincter to fully relax after a swallow. This implied an increasing ability for effective and faster swallowing coordination with increasing postmenstrual age [18]. Breathing occurs during sucking and is interrupted shortly by swallowing [19]. Timing of the swallow in the respiratory phase differs in newborns and preterm infants and changes with increasing PMA. During sucking and prolonged breathing pauses are common in preterm infants [6, 7, 20]. These can be caused by an inability to stop continuous suck–swallow sequences or multiple swallowing events [11, 21], as well as laryngeal chemoreflexes in response to milk contacting the larynx [22]. These periods may lead to desaturations, bradycardia, and early fatigue [4, 9, 11, 23]. Oral feeding skills do not mature synchronously, and the necessary coordination for safe and successful feeding may not be well-established even beyond corrected term age [9, 13]. Instrumental-based swallow studies such as endoscopic or videofluoroscopic swallowing study (VFSS) have detected silent aspirations in preterm infants even close to term age [10, 12, 24].

The underlying reasons of aspirations are not sufficiently known. It is unclear which aspects of the isolated functions of sucking, swallowing, and breathing and/or which part of their coordination is responsible for the risk of aspiration. Other than Rommel et al. [18] most research has not reported on further differentiation of the swallowing process but focused on the identification of the incidences of swallowing in the suck–swallow–breathe sequence. For these measures, visual sight, acoustic signals, surface drums or electromyography were used as well as measurements of pressure changes at the back of the pharynx using specific transnasal tubes [6, 7, 15, 16, 20, 21, 25–27].

To the authors’ best knowledge instruments are missing for newborns and infants that are non-invasive, easy to use, applicable in breast and bottle feeding without disturbing the swallowing process and in addition do not expose infants to any kind of radiation. Ideally instruments need to be suitable to differentiate the swallowing process in its unique aspects and can be combined with measuring breathing for identification of swallow–breathe coordination.

The purpose of the present study was to examine whether it is possible to investigate the swallowing process in neonates and preterm infants using the combined bioimpedance (BI) and electromyography (EMG) (BI/EMG) measurement device. Testing was performed to determine whether preterm infants’ hyoid and larynx excursions were smaller and muscle activity weaker during the swallowing process and coordination with breathing less optimal. Differences may then be indicative for possible aspirations.

## Patients and methods

### Study subjects

Healthy newborns and preterm infants born at the University Children’s Hospital Düsseldorf were recruited for a single measurement. Newborns were measured during a regular breast or bottle feed at the age of 4–8 weeks. Exclusion criteria were acute or chronic diseases, orofacial anomalies that may influence sucking or swallowing mechanism, and prematurity. Preterm infants cared for at the University Children’s Hospital Düsseldorf were recruited for a single bottle feed at 34–35 week postmenstrual age (PMA). The same exclusion criteria were applied with the addition of the necessity of breathing support (continuous positive airway pressure (CPAP), high flow nasal cannula (HFNC)). Preterm infants were required to have at least 4 days of feeding experience and needed to be able to take at least 15 ml orally during the feeds. All parents were informed of the procedure and intention of the study and gave written consent to participate. The study was approved by the ethics committee of the University on Jan. 8th, 2016, registration number 2015094347.

### Combined BI/EMG measurement device

The research device measured BI/EMG during suck–swallow bursts. In conjunction with a respiratory belt, which measured depth of chest movements and breathing pauses, the coordination of pharyngeal processes with respiration was recorded.

### The BI/EMG measurement system

The measurement system (RehaIngest^®^, Hasomed, Magdeburg) applies an alternating current across the electrodes placed on the sternocleidomastoid muscles for bioimpedance measurement. The amplitude of the current is below the perception threshold. Using surface electrodes for EMG measurement, a signal from several muscles is obtained by superimposing the muscles. Surface electrodes take up myoelectric action potentials of several muscles that are superimposed in the EMG measurement. Different frequency ranges for EMG and BI enable combined measurement of BI and EMG with one pair of electrodes. The BI signals reflect changes in pharyngeal tissue [28]. Further studies in adults specified that typical changes in the BI signal correlate with the change of position of larynx and hyoid [29, 30]. These results were verified by correlating the measurement curves with excursion of the larynx and the hyoid determined with videofluoroscopy [29]. Videofluoroscopic studies in adults have shown that hyoid movement correlates with the extent of pharyngeal closure [31–33]. The EMG signal provides additional information about the onset and extent of muscular activity of the tongue and floor of the mouth. The origin of the measurement signals is explained as: “Bioimpedance is a complex resistance parameter defined as the ratio of voltage to current arising across an electrical conductor. The ratio of voltage to current in the pharyngeal cavity changes depending on activity (swallowing/breathing, (…)). During breathing, the pharyngeal cavity is open and filled with air. Since air is a poor conductor, its electrical resistance is high. During a swallow, the pharyngeal cavity narrows as a result of the upward/forward movement of the hyoid and larynx. Electrical resistance falls, as the space is filled with tissue. Tissue is a good electrical conductor.” [30, p.2].

The studies by Nahrstaedt et al. [28] and Schultheiss et al. [29] present BI/EMG-based features that represent typical anatomical changes in adult swallowing. These features were verified by simultaneous videoflouroscopy [29].

**Fig. 1:**
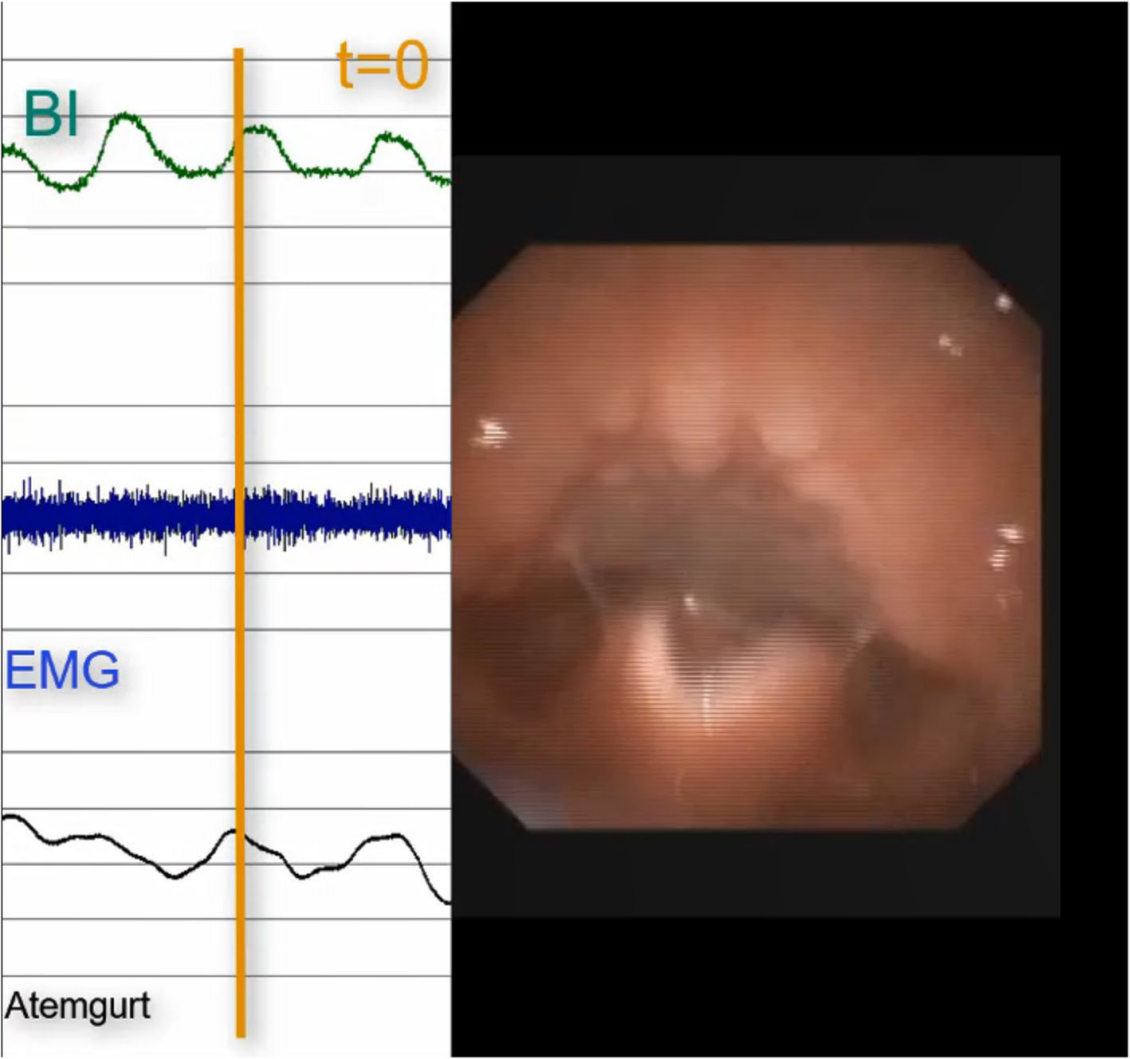
Endoscopic view; inhalation, open larynx/pharyngeal opening, bioimpedance high*. BI* bioimpedance, *EMG* electromyography, “Atemgurt” respiratory belt

**Fig. 2:**
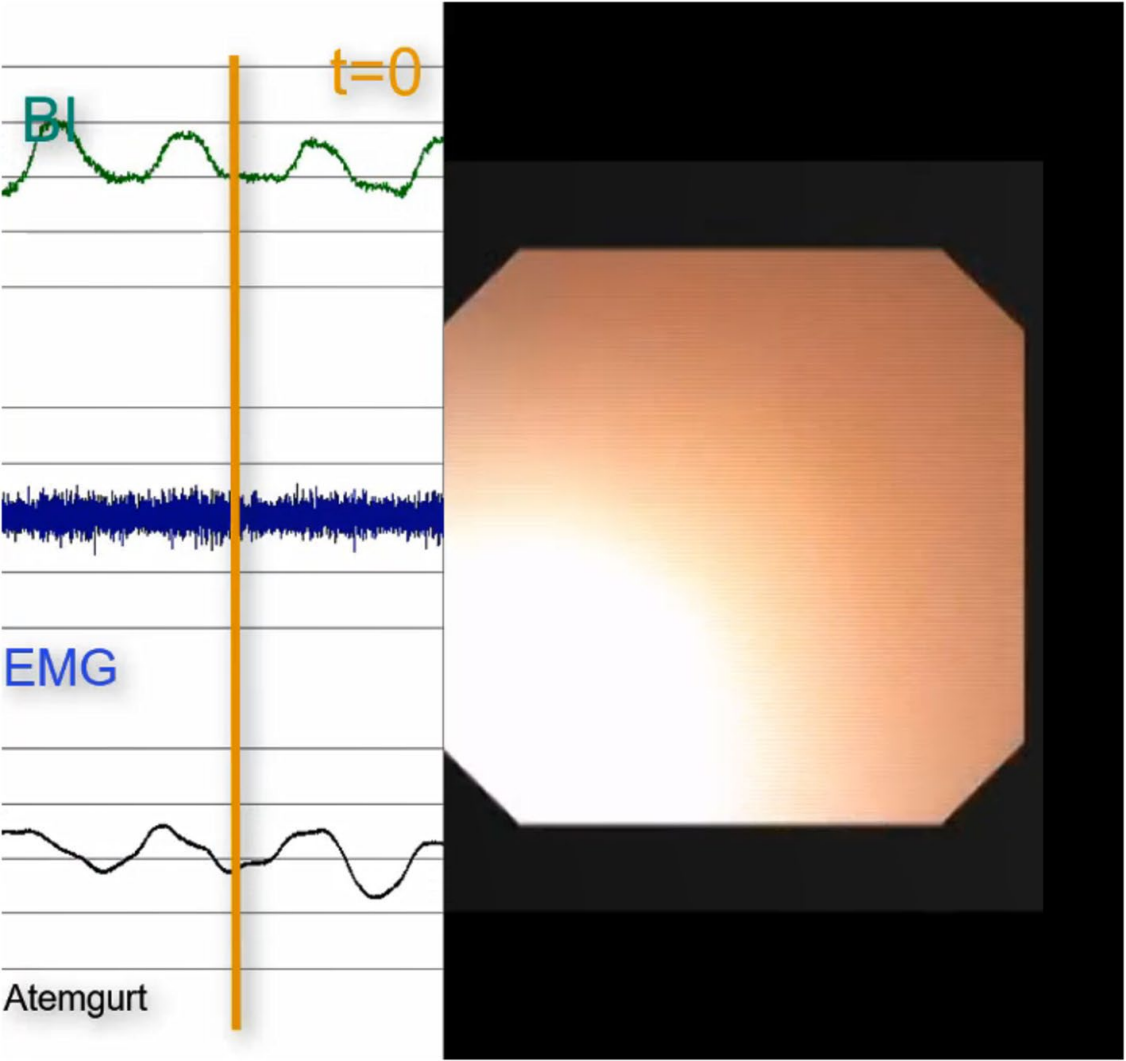
Endoscopic „white out“, pharyngeal closure, bioimpedance dropped to minimum, short breathing pause

**Fig. 3:**
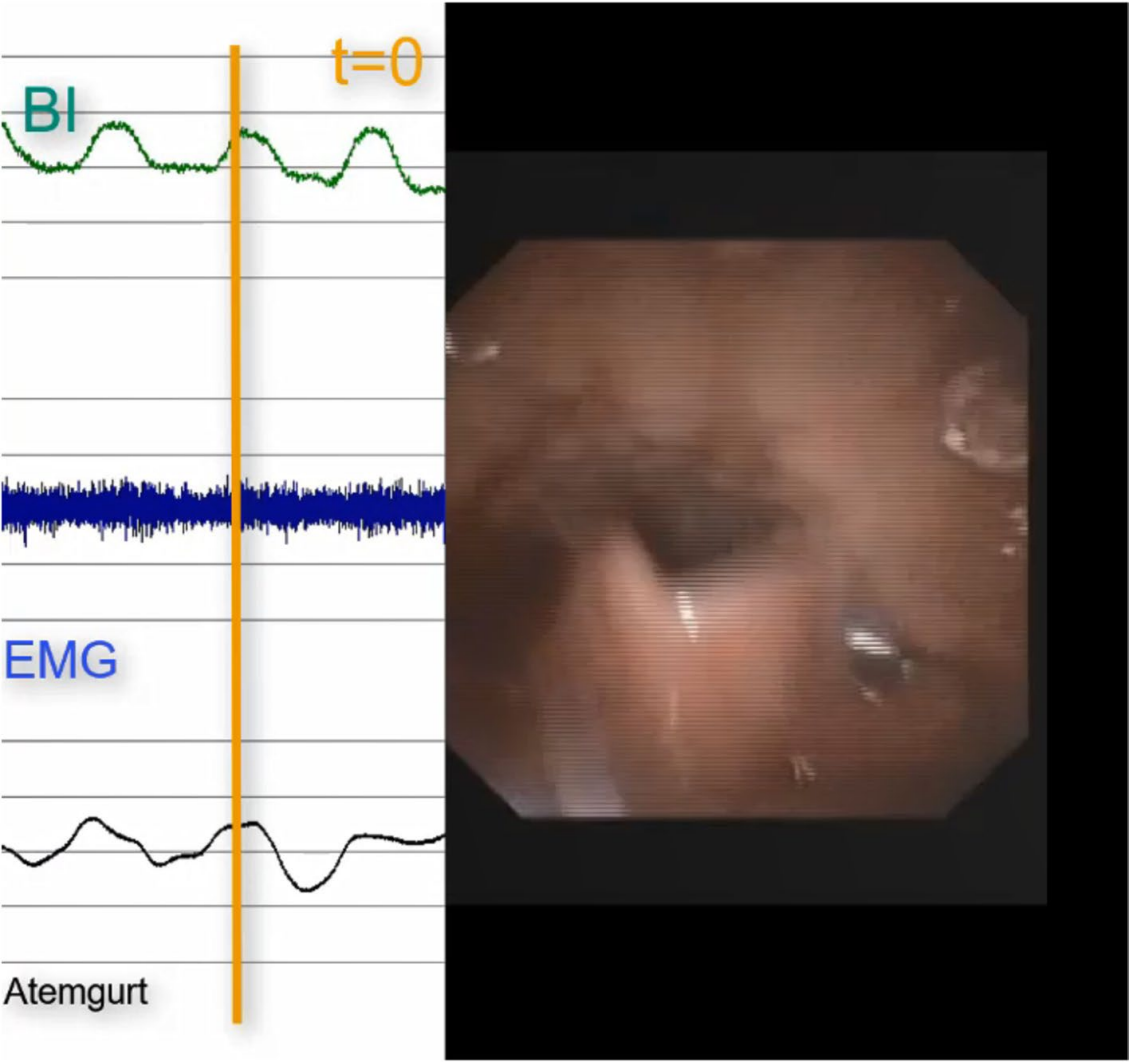
Endoscopic pharyngeal re-opening, exhalation, bioimpedance back at initial level

Endoscopic and video fluoroscopic examinations in children show a comparable pattern of movement of the tongue base and larynx during food intake as in adults, although these specific differentiations have not yet been validly tested in the absence of suitable examination instruments [34, 35]. In addition, in children there is an approach of tongue base to the posterior pharyngeal wall during elevation of the larynx, causing an occlusion of the pharynx resulting in the “white out phase” during endoscopic examination. This was confirmed in combined examinations of endoscopic examination and RehaIngest^®^. Figures 1, 2 and 3 show the endoscopic view of the larynx and synchronous measurement of BI/EMG and breathing during a swallowing process. Figure 1 shows larynx/pharyngeal opening and high bioimpedance with inhalation. Figure 2 shows typical drop to a bioimpedance minimum with pharyngeal closure and endoscopic “white out” as well as a short breathing pause. Figure 3 shows pharyngeal re-opening, exhalation and the bioimpedance at the initial high level.

Based on clinical experience and these investigations, it can be assumed that there is a modification in pharyngeal tissue and space occurring during swallowing which can be registered by the change in the bioimpedance signal comparable to adults. Thus, swallows could be detected based on the typical change in bioimpedance curve. The change in bioimpedance corresponds to the velocity and extent of pharyngeal closure. If related to the change in respiratory curves, the coordination could be described.

This device allows non-invasive measurements of the swallowing process and can be combined with measurement of breathing. No radiation exposure or invasive procedure is necessary. Therefore, we considered this instrument suitable to pursue our research question in the neonatal and preterm group.

### Study protocol

Approximately 30 min prior to their regular feed the respiratory belt was positioned around the thorax and five electrodes were placed on the babies’ skin: two electrodes at both sides laterally above the larynx, two electrodes at onset of sternocleidomastoid muscle, and one reference electrode on one of the cheeks. The electrodes were connected to BI/EMG measurement device, which was connected to a laptop recording the data. At the time of the feed, the newborns and preterm infants needed to be awake and ready to feed. Preterm infants received no interventions or examinations the hour before the feed to allow for optimal state for feeding.

### Annotation of swallowing and breathing

The data from the measurement module (signals from BI/EMG and respiration) was continuously displayed on a self-developed measurement program on a computer (Windows 10) during the measurement and saved in EFD format. For further analysis, the data were exported and processed in an EDF browser (https://www.windows10download.com/edfbrowser/23.11.2022).

For further analysis, the beginning and end of swallows as well as the beginning and end of respiratory movements had to be marked in the measurement curves by annotations. In adults, the marking of swallows is unambiguous. A swallow starts with an EMG activity, the posterior tongue is lifted, then a rapid decrease of the BI curve follows which corresponds to a laryngeal lift. In this case, the beginning and end of laryngeal elevation is marked in the change of the BI curve [29]. In the case of respiration, the turn of the respiratory curve (rise and fall of the respiratory curve) describing inhalation or exhalation is marked, as well as the breath stop.

The marking of the BI/EMG measurements had to be adapted to the different swallowing behavior of the neonatal/preterm population. In this age group, there usually is a continuous suck–swallow sequence with consecutive sucking and swallowing and thus almost continuous EMG activation. Sucking is described as a back-and-forth movement of the tongue as well as lowering of the tongue for increasing the intraoral vacuum [36, 37]. The backward/downward movement of the base of the tongue allows transporting of milk into the pharynx, subsequently the pharynx must close to initiate the swallowing process. In the collected measurement data, sucking and swallowing could not be separated, because sucking and swallowing merge into each other and have comparable activities in BI/EMG. For this reason, an adjustment of the annotations was necessary:

Sucking was defined as a maximum of a bioimpedance with simultaneous EMG activity before a drop in the BI curve occurred. During sucking the pharynx is still open and filled with air, for this reason the BI curve is found on a maximum plateau.

The first drop of the BI curve after such a phase was marked as the start of a swallow. For a swallow, the tongue is moved backwards, and the larynx is lifted with the following constriction of the pharynx. This is shown in the BI curve as a drop in the measurement signal. The end of the swallowing phase was defined as the rebound of the BI curve to 3/4 of the starting point.

**Fig. 4:**
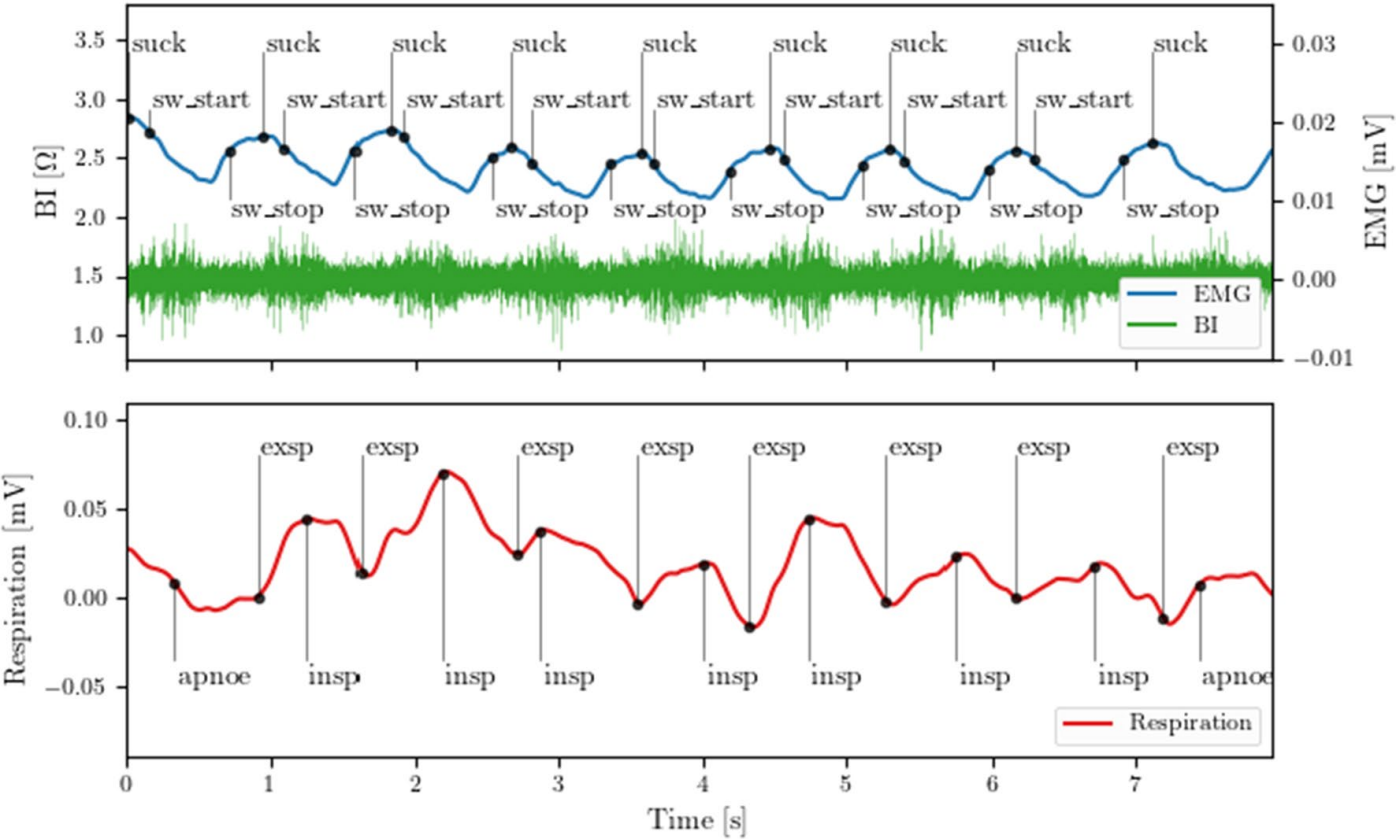
Annotations of swallowing and breathing. *suck* start of sucking*, sw_start* start of swallowing process, *sw_stop* end of swallowing process. *EMG* electromyography, *BI* bioimpedance, *apnoe* breathing pause, *exsp* exspiration, *insp* inspiration

For the present study, annotations were marked for sucking, the beginning and end of swallowing, and the beginning of inhalation, exhalation, and pauses in breathing (Fig. 4). A breathing pause had to be at least three seconds long to be considered. We decided to consider a suck–swallow burst only if BI/EMG activation were continuously present during at least three simultaneous swallows. No annotations were marked outside of suck–swallow bursts. All annotations were marked by trained observers and reviewed by a second observer. When conflicts arose, consensus was reached and annotations were modified accordingly, if necessary.

The EDF files with the marked traces were then read by a Python script and exported to a CVS file for further calculation.

The swallow features introduced by Schultheiss et al. [29] were adapted to assess the swallowing process in NB and PT. Extracting the features from BI data $BI^{n}$, EMG data $EMG^{n}$, and respiration data $RES^{n}$ with index $n$ utilizes the start time $t_{{{\text{BI}}}}^{{{\text{start}}}}$, and end time $t_{{{\text{BI}}}}^{{{\text{end}}}}$ of BI valleys, and the start times of inspiration $t_{{{\text{RES}}}}^{{{\text{insp}}}}$, and expiration $t_{{{\text{RES}}}}^{{{\text{exsp}}}}$ provided by the manual annotations according to Fig. 4.

The BI, EMG, and respiration measurements were filtered prior to the feature extraction. A third order Butterworth low-pass filter with 10 Hz cutoff frequency reduces the noise in BI and respiration measurements. Applying a third order Butterworth high-pass filter with 15 Hz cutoff frequency removes the offset of EMG measurements. Determining the smallest value between $t_{{{\text{BI}}}}^{{{\text{start}}}}$ and $t_{{{\text{BI}}}}^{{{\text{end}}}}$ yields the minimal value ${\text{BI}}^{{{\text{min}}}}$ and timepoint $t_{{{\text{BI}}}}^{{{\text{min}}}}$ of a BI valley.

The feature extraction deploys the following definitions:

- Duration of a swallow $t_{{{\text{sw}}}} = t_{{{\text{BI}}}}^{{{\text{end}}}} - t_{{{\text{BI}}}}^{{{\text{start}}}} { }$ is defined as the time span from the start time $t_{{{\text{BI}}}}^{{{\text{start}}}}$ to the end time $t_{{{\text{BI}}}}^{{{\text{end}}}}$ of a swallow valley.
- The velocity of laryngeal excursion $S_{{{\text{BI}}}} = ({\text{BI}}^{{{\text{start}}}} - {\text{BI}}^{{{\text{min}}}} )/\left( {t_{{{\text{BI}}}}^{{{\text{min}}}} - { }t_{{{\text{BI}}}}^{{{\text{start}}}} } \right)$ reflects the steepness of the BI drop during the laryngeal elevation by calculating the slope of a line from the BI valleys start value ${\text{BI}}^{{{\text{start}}}}$ at timepoint $t_{{{\text{BI}}}}^{{{\text{start}}}}$ to minimal BI value ${\text{BI}}^{{{\text{min}}}}$ at timepoint $t_{{{\text{BI}}}}^{{{\text{min}}}}$.
- The extent of laryngeal movement $\Delta_{{{\text{BI}}}} = {\text{BI}}^{{{\text{start}}}} - {\text{BI}}^{{{\text{min}}}}$ is the difference of start BI value ${\text{BI}}^{{{\text{start}}}}$ and the minimal BI value ${\text{BI}}^{{{\text{min}}}}$ of a BI swallow valley.
- The extent of pharyngeal closure is the area $A_{{{\text{BI}}}} = \mathop \sum \nolimits_{{n = n_{{{\text{BI}}}}^{{{\text{start}}}} }}^{{n_{{{\text{BI}}}}^{{{\text{min}}}} }} \frac{1}{{f_{s} }}\left( {{\text{BI}}^{n} - {\text{BI}}^{{{\text{min}}}} } \right)$ under the decreasing BI curve from BI valley’s start sample at index $n_{{{\text{BI}}}}^{{{\text{start}}}} = t_{{{\text{BI}}}}^{{{\text{start}}}} f_{s}$ to the minimal sample at index $n_{{{\text{BI}}}}^{{{\text{min}}}} = t_{{{\text{BI}}}}^{{{\text{min}}}} f_{s}$ with $f_{s}$ denoting the sample frequency of the BI/EMG data.
- The EMG maximum ${\text{EMG}}^{{{\text{max}}}} = \mathop {\max }\limits_{{n_{{{\text{BI}}}}^{{{\text{start}}}} \le n \le n_{{{\text{BI}}}}^{{{\text{min}}}} }} \left( {{\text{EMG}}^{n} } \right)$ is the greatest value of the EMG data during the BI drop of BI valley from $n_{{{\text{BI}}}}^{{{\text{start}}}}$ to $n_{{{\text{BI}}}}^{{{\text{min}}}}$.
- The EMG activity $\sigma^{{{\text{EMG}}}} = \sqrt {\frac{1}{{n_{{{\text{BI}}}}^{{{\text{min}}}} - n_{{{\text{BI}}}}^{{{\text{start}}}} }}\mathop \sum \nolimits_{{n = n_{{{\text{BI}}}}^{{{\text{start}}}} }}^{{n_{{{\text{BI}}}}^{{{\text{min}}}} }} \left( {{\text{EMG}}^{n} - \overline{{{\text{EMG}}}} } \right)^{2} }$ is defined as the standard deviation of the EMG data during the BI drop from $n_{{{\text{BI}}}}^{{{\text{start}}}}$ to $n_{{{\text{BI}}}}^{{{\text{min}}}}$ with $\overline{EMG}$ representing the mean of the EMG data.
- The respiration duration considers the time spans of inspiration $\Delta t_{{{\text{RES}}}}^{{{\text{insp}}}} = t_{{{\text{RES}}}}^{{{\text{insp}}}} - t_{{{\text{RES}}}}^{{{\text{exsp}}}}$, and the time spans of expiration $\Delta t_{{{\text{RES}}}}^{{{\text{exsp}}}} = t_{{{\text{RES}}}}^{{{\text{exsp}}}} - t_{{{\text{RES}}}}^{{{\text{insp}}}}$ computed from the start timepoints of inspiration $t_{{{\text{RES}}}}^{{{\text{insp}}}}$, and expiration $t_{{{\text{RES}}}}^{{{\text{exsp}}}}$.
- The depth of thoracic expansion includes inspiration $\Delta^{{{\text{RES}}}} = {\text{RES}}^{{{\text{insp}}}} - {\text{RES}}^{{{\text{exsp}}}}$ and expiration $\Delta^{{{\text{RES}}}} = {\text{RES}}^{{{\text{exsp}}}} - {\text{RES}}^{{{\text{insp}}}}$ movements using the values of the respiration data at the start of inspiration ${\text{RES}}^{{{\text{insp}}}}$ and expiration ${\text{RES}}^{{{\text{exsp}}}}$.

Finally, the mean and standard deviation of each feature from a swallow–suck burst was calculated. The mean feature values are the basis for statistical analysis.

### Statistical analysis

The data were statistically evaluated using SPSS Statistics (IBM SPSS Statistics 25, 2021). To compare swallowing and breathing performance of newborns and preterm infants we calculated mean values (M) and standard deviations (SD) of the different features of the swallowing process and breathing for the whole feed and for the first two suck–swallow bursts. The exact version of the Mann–Whitney *U* test (one-tailed) was used to test for group differences between newborns and preterm infants with regard to breathing patterns and the swallowing process. *P* value was set at level of 0.05.

## Results

### Subject characteristics

Forty newborn infants were measured, of whom 15 were bottle-fed and 25 were breastfed. Mean age at measurement of newborns was 6.7 weeks (w) (SD 0.9w, range 4.4–8.7w).

Twenty preterm infants were measured during a bottle feed. Mean gestational age at birth was 30.5 weeks (SD: 2.1w, range: 25.9–32.9w) (Table 1). The preterm infants were healthy: none had a history of intraventricular hemorrhage (IVH) or periventricular leukomalacia (PVL), and none were small for gestational age. Their mean age at time of measurement was 35.1w PMA (SD: 0.4w, range: 34.4–35.7w). All infants met inclusion criteria.

**Table 1:** Characteristics of the subjects

	Gestational age at birth	Birthweight	Age (PMA) at measurement	Weight at measurement
	Weeks	Gram	Weeks	Gram
Newborns	Term born	M 3425.5 (SD 458.3)	M 6.7 (SD 0.9)	M 4595.1 (SD 583.5)
Preterm infants	M 30.5 (SD 2.1)	M 1449.5 (SD 388.6)	M 35.1 (SD 0.4)	M 2081 (SD 262)

Listed are mean value (M) and standard deviation (SD)

The recruiting period was 07/2016–11/2017 for newborn infants and 02/2020–02/2021 for preterm infants due to available resources. Measurements with BI/EMG measurement device and the respiratory belt did not restrict the children’s movement or milk intake. The measured signals were transferred to the EDF browser without significant interruption. Evaluation of all data was performed from 02/2021 to 10/2021 due to available resources.

### Swallowing patterns

The specific characteristics of the swallowing process determined did not differ significantly between preterm and newborn infants over the entire feeding course (Table 2). This included no significant differences in velocity (NB – 0.005 Ω/s; PI – 0.004 Ω/s, *p* = 0.09) and none in the extent of pharyngeal closure (NB 0.069 Ωs; PI 0.066 Ωs, *p* = 0.17). Preterm infants showed a comparable pattern of pharyngeal closure compared to neonates.

**Table 2:** Parameters of laryngeal movement for the whole feed

	Velocity of larynx elevation	Extent of laryngeal movement	Extent of pharyngeal closure	Duration of swallow
	$S_{{{\text{BI}}}}$	$\Delta_{{{\text{BI}}}}$	$A_{{{\text{BI}}}}$	$t_{{{\text{sw}}}}$
Newborns	− 0.005 (SD 0.003)	0.306 (SD 0.287)	0.069 (SD 0,70)	0.553 (SD 0,079)
Preterm infants	− 0.004 (SD 0.004)	0.257 (SD 0.106)	0.066 (SD 0.029)	0.693 (SD 0.071)
p value	0.09	0.72	0.17	< 0.001*

Mean values and standard deviation for the whole feed

*p value significant at *p* < 0.05

Because the number and duration of suck–swallow bursts varied throughout the measurement period, an additional analysis of the first two bursts was performed to better investigate the changes in pharyngeal movement at the onset of oral intake. This revealed that in the first two suck–swallow bursts, preterm infants had a higher velocity of laryngeal elevation (NB – 0.003 Ω/s; PI − 0.004 Ω/s, *p* < 0.001) and a greater magnitude of laryngeal and pharyngeal movements than newborns (NB 0.184 Ωs; PI 0.257 Ωs, *p* = 0.004) (Table 3). The duration of the pharyngeal closure was significantly longer in preterm infants in the first two suck–swallow bursts (NB 0.375 s; PI 0.700 s, *p* < 0.001*), as well as throughout the whole feed (NB 0.553 s; PI 0.693 s, *p* < 0.001*) (Tables 2 and 3). This means preterm infants needed more time to complete the individual swallows compared to newborns.

**Table 3:** Parameters of laryngeal movement (first two suck–swallow bursts)

	velocity of larynx elevation	extent of laryngeal movement	extent of pharyngeal closure	duration of swallow
	$S_{{{\text{BI}}}}$	$\Delta_{{{\text{BI}}}}$	$A_{{{\text{BI}}}}$	$t_{{{\text{sw}}}}$
Newborns	M: − 0.003 SD: 0.002	M: 0.184 SD: 0.05	M: 0.040 SD: 0.02	M: 0.375 SD: 0.07
Preterm infants	M: − 0.004 SD: 0.001	M: 0.257 SD: 0.04	M: 0.069 SD: 0.02	M: 0.700 SD: 0.12
*p* value	< 0.001*	0.004*	< 0.001*	< 0.001*

Mean values (M) and standard deviation (SD) of first two suck–swallow bursts

*p value significant at *p* < 0.05

### EMG

Throughout the whole feed the mean maximum of EMG activity of suck–swallow bursts of NB (0.035 mV) was significantly higher than that of PI (0.011 mV) (*p* < 0.001). The mean extent of EMG activity during suck–swallow bursts was significantly greater in NB (0.009 mV) than in PI (0.003 mV) (*p* < 0.001). These differences were not significant in the first two suck–swallow bursts (mean maximum EMG NB: 0.023 mV, PI: 0.011 mV, *p* = 0.11, mean extent of EMG activation NB: 0.006 mV, PI: 0.003 mV, *p* = 0.06).

### Breathing patterns

**Fig. 5:**
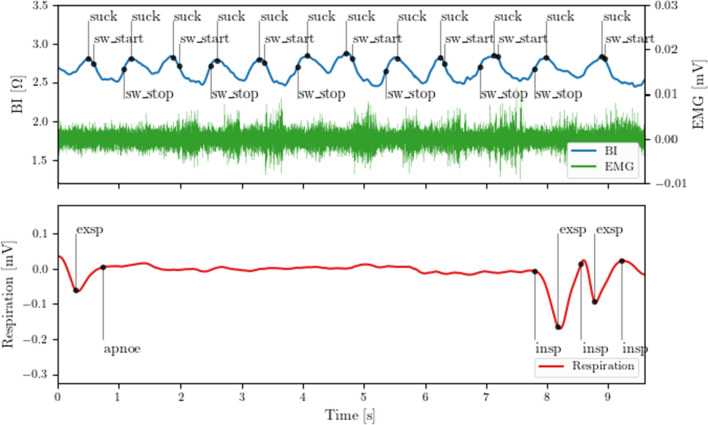
Prolonged breathing pause in a preterm infant. *suck* start of sucking*, sw_start* start of swallowing process, *sw_stop* end of swallowing process. *EMG* electromyography, *BI* bioimpedance, *apnoe* breathing pause, *exsp* exspiration, *insp* inspiration

Changes in inhalation and exhalation as well as breathing pauses could be detected well with the respiratory belt and were transcribed to the EDF browser and marked accordingly. Very short breathing pauses during swallowing could not be identified by the respiratory belt as it is not as sensitive to very brief changes in movements of the chest. Breathing patterns were different among PI and NB. Chest movements resulting from inhalation and exhalation were significantly deeper in NB (mean 0.27 mV, SD 0.11 mV) than those of PI (mean 0.122 mV, SD 0.05 mV) (*p* < 0.001). NB had fewer breathing pauses, and none exceeded 3 s. PI had frequent breathing pauses (Fig. 5): they exhibited a mean number of 7.9 breathing pauses with a duration over 3 s.

## Discussion

The aims of the investigations were to find and test a method that can record the swallowing and breathing of newborns and preterm infants and to evaluate the changes in the pharynx during the swallowing process. In addition, differences in preterm infants’ and newborns’ swallowing and breathing were to be identified.

The method used in this study measures BI/EMG via skin electrodes and allows conclusions to be drawn about muscular activity of the floor of the mouth and tongue, changes in the pharynx that correspond to movements during the swallowing process and duration of the swallowing process. The additional possibility to synchronously measure respiration allows to answer further questions. The measurements could be carried out without complications and resembled the data in adults with the described adaptation very well. The easy application of the measurement device with no disturbance of the feeding process is very promising.

Some previous examination methods have significant limitations and disadvantages. Some have low reliability (external observation) [38] and more invasive methods (e.g. pressure probes, endoscopic examinations) may alter the swallowing process [6, 12, 15, 16, 18, 20, 21]. The use of VFSS exposes infants to radiation and typical feeding position may not be possible and direct breastfeeding cannot be observed [39, 40]. Noninvasive identification of swallowing [7, 25, 26] can limit the possibility of differentiating the swallowing process itself any further. A measurement method should be able to be combined with a sensor for breath measurement. This is difficult or impossible to implement in everyday clinical practice with all the methods listed.

Radiological studies in neonates described a comparable sequence of hyoid and laryngeal movement during swallowing as in adults. Hyoid position values in children infrequently fell within the 95% confidence intervals of adults [35]. Thus, it might be reasonable to assume comparable findings in children. Verification of the bioimpedance measurement method in newborns with videofluoroscopy could not be successful, because the electrodes overlay the relevant anatomical regions in the videofluoroscopy and the hyoid and larynx can no longer be reliably detected. Recognition of the hyoid in newborns is problematic [35].

With this novel research device differences in the swallowing process of newborns and preterm infants were to be detected. We hypothesized preterm infants to show swallowing movements of less velocity and extent compared to neonates that contribute to the described incoordination in feeding and risk for aspiration. When the entire period of feeding is examined for the parameters of velocity and extent of pharyngeal closure, there is no statistical difference. If the first two suck–swallow bursts are considered, velocity and extension of pharyngeal closure is significantly faster and greater in preterm infants than in neonates. However, the length of a swallow, i.e., the period until a new swallow is started, is significantly longer in preterm infants.

We did not expect preterm infants to show comparable abilities as newborns and not at all a higher velocity and greater extent of pharyngeal closure in the first two suck–swallow bursts compared to neonates. Due to the lower weight and thus lower muscle mass, we expected the preterm infants’ swallow to show less precise, slower, and fewer laryngeal movements leading to less pharyngeal closure.

Preterm infants, on the other hand, showed comparable muscle activation at the beginning of feeding. The extent and velocity of laryngeal movement was higher than in neonates. A possible explanation for this behavior may be found in an immature neurological control of the swallowing movement.

Whereas in newborns a complete sensory-motor control circuit already regulates the extent and sequence of the swallowing movement, this control circuit is not yet fully developed in preterm infants. As a result, there is an excessive movement at the start of the swallowing sequence due to limited sensory-motor control. This can also lead to disturbances at the beginning of feeding. Throughout the feeding muscle activation was lower in preterm infants but pharyngeal closure remained comparable. Apparently essential components of a safe swallowing process including fast and decent pharyngeal closure are adequately developed in preterm infants at least at the postmenstrual age of 34/35 weeks.

Although measurements, technique, and focus differed, the data are somewhat comparable to research by Rommel and colleagues [18]. While the present measurement was directed toward changes in the pharyngeal closure, Rommel et al. [18] had measured pharyngeal pressure and upper esophageal sphincter (UES) closure. They found no change in either the velocity of pharyngeal peristalsis or the amplitude of peak pharyngeal pressure in preterm infants from 32 to 36 week PMA during swallowing. This, like our own data, indicates an identical course of pharyngeal closure during swallowing as early as 32 week PMA. A more detailed analysis of peak pharyngeal pressure revealed a significantly lower pressure of 1 cm above UES in preterm infants of 31/32 week PMA [18]. Because pressure development in the pharynx is dependent on the force present during swallowing, these results are comparable to prolonged swallowing in preterm infants. In our data we identified prolonged swallowing of preterm infants at a slightly older postmenstrual age of 34/35 weeks compared to neonates. Rommel et al. [18] also noted a further difference in timing during the swallowing process. Younger preterm infants took more time to fully relax the upper esophageal sphincter compared to infants of corrected term age. If the opening movement of the upper esophagus depends on the extent and velocity of laryngeal elevation, this delay corresponds to the measured prolonged swallowing in our study [41]. With increasing PMA, the variability of this relaxation time decreased, suggesting greater consistency. Rommel et al. [18] concluded that swallow coordination in infants of near-term age is most effective when the timing of pharyngeal contraction and pressure and function of the UES are well-matched. A lack of timing at younger postmenstrual ages is thought to make aspiration possible [18].

Despite the comparable swallowing patterns, studies show that preterm infants are at higher risk of aspirating during feeding. The reasons are not yet fully understood. In our own study, three significant differences were seen between the groups: (1) muscle activation, and thus the number of active muscle fibers, decreased during the swallowing process after the first two suck–swallow bursts in preterm infants, (2) the duration of the single swallow was longer in preterm infants and (3) the respiratory activity is lower in preterm infants. Whereas newborns did not exhibit any pauses in breathing longer than 3 s, the number of these pauses was significantly greater in preterm infants with significantly fewer respiratory excursions. To our knowledge, these three changes have not yet been described.

We hypothesize that differences in muscle strength, timing, and duration of the swallowing process, as well as early fatigue during feeding and breathing, are critical factors in the development of aspiration. The longer a swallowing phase lasts, the greater the chance of exhaustion and thus for pauses in breathing. These pauses in breathing must be interrupted by inhalation to maintain oxygenation. If milk flow continues because of a mismatch between sucking and swallowing, the fluid in the throat is inhaled, which would lead to aspiration or at least penetration. Low muscle strength could exacerbate the problem by decreasing the quality, velocity, and efficiency of swallowing movements. These are also the findings from instrumental studies that described ineffective swallowing and post-swallow residue as a cause of aspiration in preterm infants [12].

In summary, increasing inefficiency of the longer swallowing process due to decreased muscle strength and premature fatigue should be considered as probable causes of increased aspiration risk in preterm infants. Thus, efforts must be made to interrupt this mechanism. More time is needed for the preterm infant to complete the swallowing process and regular breathing must be maintained. Interventions such as positioning in elevated side-lying, thickening of milk, and feeding techniques such as pacing allow more time for completing the swallow and aim to prevent infants from reaching a state of exhaustion that can lead to respiratory compromise [11, 42, 43]. These interventions will be tested in further studies.

### Limitations

The present study has several limitations. The measurement system was applied for the first time in neonates and preterm infants in our study; therefore, comparable data in this age group is lacking. There are differences between adults and infants that also relate to the dimensions of the anatomy involved in the swallowing process. In addition, suck–swallow sequences are continuous compared with single swallows in adults. Basically, each swallowing process in both groups involves closure of the pharynx by an upward movement of the larynx to prevent entry of food or fluid into the larynx. Thus, transmission of the processes appears to be possible, although not in as much detail as in adults.

When respiration is measured by an abdominal or chest belt, it is recorded with a significant delay. The breath has already passed the larynx, the site to be observed, by the time the measurement can detect a change. A more accurate measurement would have been possible in using nasal cannulas or by placing invasive probes, i.e., a flow probe in the larynx. This interventional procedure has not been used in children.

The patient group investigated in this pilot study is small and comes from only one hospital. Further studies now need to be multicenter with larger patient groups.

## Conclusion

The measurement method used for the first time in this study enables the recording and evaluation of continuous swallowing and breathing patterns during feeding in newborns and preterm infants without restricting or stressing the swallowing process or the infant. Our data shows no differences in critical aspects of the swallowing process between the groups studied: extension and velocity of pharyngeal closure throughout the whole feed are comparable in both groups. The differences between the groups appear to be due to differences in muscle strength that negatively affect the timing and duration of swallowing and respiration. The preterm infant fatigues more quickly. This is why the quality, efficiency, and safety of the coordination of swallowing and breathing deteriorates at an earlier stage in the feeding of preterm infants. Further studies are needed to determine which adapted feeding methods can minimize these differences to support safe and efficient feeding of preterm infants.

## Data Availability

The data sets generated during either and/or analyzed during the current study are not publicly available due to limited license of the data for the current study but are available from the corresponding author on reasonable request.
